# Applications and Prospects of Artificial Intelligence-Assisted Endoscopic Ultrasound in Digestive System Diseases

**DOI:** 10.3390/diagnostics13172815

**Published:** 2023-08-30

**Authors:** Jia Huang, Xiaofei Fan, Wentian Liu

**Affiliations:** Department of Gastroenterology and Hepatology, Tianjin Medical University General Hospital, No. 154, Anshan Road, Heping District, Tianjin 300052, China; helga@tmu.edu.cn (J.H.); ffan@tmu.edu.cn (X.F.)

**Keywords:** artificial intelligence, endoscopic ultrasound, machine learning, deep learning, digestive system diseases

## Abstract

Endoscopic ultrasound (EUS) has emerged as a widely utilized tool in the diagnosis of digestive diseases. In recent years, the potential of artificial intelligence (AI) in healthcare has been gradually recognized, and its superiority in the field of EUS is becoming apparent. Machine learning (ML) and deep learning (DL) are the two main AI algorithms. This paper aims to outline the applications and prospects of artificial intelligence-assisted endoscopic ultrasound (EUS-AI) in digestive diseases over the past decade. The results demonstrated that EUS-AI has shown superiority or at least equivalence to traditional methods in the diagnosis, prognosis, and quality control of subepithelial lesions, early esophageal cancer, early gastric cancer, and pancreatic diseases including pancreatic cystic lesions, autoimmune pancreatitis, and pancreatic cancer. The implementation of EUS-AI has opened up new avenues for individualized precision medicine and has introduced novel diagnostic and treatment approaches for digestive diseases.

## 1. Introduction

**Figure 1:**
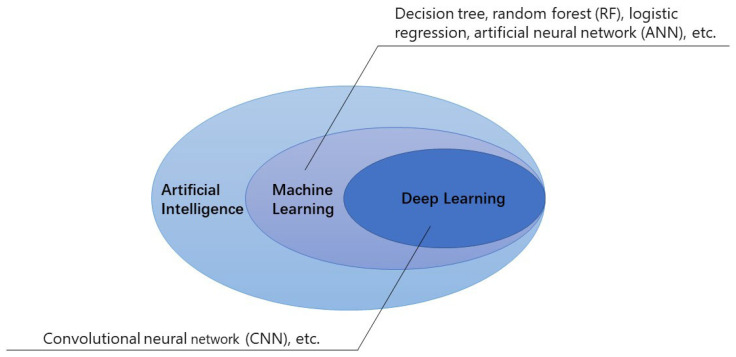
Machine learning and deep learning have emerged as prominent AI architectures extensively employed in the field of medicine. Figure 1 presents several typical examples of techniques that can be effectively applied to these two architectures.

Artificial intelligence (AI) is a computer program that has been developed by humans to mimic the abilities of the human brain to think, judge, and react. Since the term “AI algorithm” was first proposed by John McCarthy in 1956, it has undergone a significant transition from artificial narrow intelligence (ANI) to artificial general intelligence (AGI). While ANI refers to AI that is trained to focus on performing specific tasks, AGI is a theoretical form of AI that possesses self-awareness and the ability to solve problems, learn, and plan for the future. Artificial super intelligence (ASI) surpasses human intelligence and capabilities, but it is still in a completely theoretical stage. The realization of ASI is the ultimate purpose of AI research. Currently, machine learning (ML) and deep learning (DL) are the main architectures of AI that are widely used in medical image recognition. ML is a computer program that enables machines to learn without explicit programming. It could quantify the features of medical images based on inherent regular patterns. Various ML technologies, such as decision trees, random forest (RF), logistic regression, and artificial neural networks (ANN), are commonly employed in diagnosing medical images. DL is a new field of ML, which is realized by imitating the mechanisms of the human brain in three phases: data collection and annotation, construction of DL architecture, and training and verification of its capabilities. One of the most notable advantages of DL is its ability to automatically detect and objectively identify features of interest in medical images. Compared with ML, DL is easier to implement and offers higher accuracy. Convolutional neural network (CNN) is commonly used in the field of DL, and it is considered one of the best-performing algorithms for recognizing images at present [1,2,3] (Figure 1). In recent studies, the focus has been on evaluating the diagnostic performance of individual DL models for various diseases. However, each model may have its own unique ability to outperform other models in feature learning. Consequently, some researchers have begun exploring the possibility of enhancing diagnostic accuracy through the integration of multiple models after model fusion. Gunasekaran et al. proposed an ensemble model (GIT-NET) that combines pre-trained ResNet50, DenseNet201, and Inception v3 models. By utilizing these models to extract features from the KVASIR v2 dataset with eight classes of digestive diseases, the authors achieved accuracies of 92.96% and 95.00% through model averaging and weight averaging methods, respectively, surpassing the baseline models [4]. Similarly, Ramamurthy et al. introduced an approach that employed a pre-trained EfficientNet B0 backbone and custom CNN (Effimix) to automatically classify gastrointestinal diseases, employing the HyperKvasir benchmark dataset. By leveraging the combined features of these two networks, the proposed model achieved an accuracy of 97.99%, along with an impressive F1 score, precision, and recall values of 97%, 97%, and 98%, respectively [5]. Zhao et al. developed an improved YOLOX model that incorporated YOLOX, group normalization, and video adjacent-frame association to achieve real-time detection of endometrial polyps. The improved model had sensitivities of 100% and 92.0% for lesions in both the internal and external test sets, surpassing the per-lesion sensitivities of the original YOLOX model, which stood at 95.83% and 77.33%, respectively. These findings highlight the potential of the improved model in reducing the risk of endometrial cancer by effectively identifying and localizing endometrial polyps [6].

In the early stages of the disease, patients often lack obvious clinical signs and symptoms, making it challenging to diagnose. Traditional imaging techniques, such as ultrasonography (US), computed tomography (CT), and magnetic resonance imaging (MRI), may not be able to detect smaller lesions. However, with advancements in gastrointestinal endoscopy technology, the potential advantages that endoscopic ultrasound (EUS) might have over other imaging modalities has been discovered. Yoshida et al. observed that EUS exhibited a median sensitivity of 93–94% in detecting pancreatic lesions, surpassing the sensitivity of magnetic MRI at 67% and CT at 53% (for detecting lesions <30 mm, *n* = 49) [7]. EUS combines ultrasound technology with endoscopic visualization, allowing for high-resolution and real-time visualization of the digestive tract lumen, obtaining ultrasonic images of the gastrointestinal tract and adjacent organs, and providing insights into the depth of tumor invasion and the presence of enlarged lymph nodes. As a result, EUS has become an important detection tool for various digestive diseases, enhancing the ability to accurately assess the nature and scope of lesions and improving the detection rate of the diseases [8,9].

**Figure 2:**
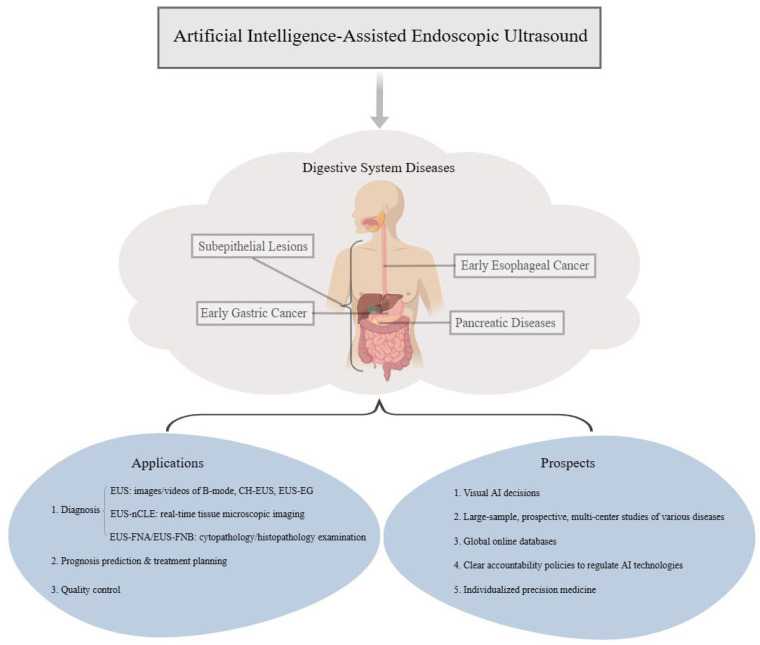
The applications of EUS-AI in gastrointestinal and pancreatic diseases, including identifying and evaluating lesions, prediction of prognosis, treatment planning, and quality control in the diagnostic process of the diseases. Figure 2 also point out the broad prospects of EUS-AI.

The diagnostic accuracy of EUS is closely related to the knowledge, experience, and operation level of the endoscopists. It is subjective to a certain extent. Some diseases are challenging to diagnose with EUS alone [10]. AI has the ability to process large amounts of data with high accuracy, and when combined with EUS, it offers objective, simple, and rapid examination. Tonozuka et al. developed a computer-aided diagnosis (CAD) system utilizing EUS images and assessed its efficacy in distinguishing between pancreatic ductal adenocarcinoma (PDAC), chronic pancreatitis (CP), and individuals with normal pancreatic conditions. The findings of this study showcased the exceptional performance of the CAD system for detecting PDAC, with a sensitivity of 92.4% and a specificity of 84.1%, respectively [11]. In a study conducted by Wang et al., the diagnostic value of single endoscopy and artificial intelligence-assisted endoscopic ultrasound (EUS-AI) in early esophageal cancer and precancerous lesions was compared. The researchers also described the diagnostic accuracy of two different models; namely, the CNN model and the Cascade region-convolutional neural network (Cascade RCNN) model. The findings revealed that the Cascade RCNN model, which was based on the soft-NMS intelligent algorithm, exhibited better diagnostic performance compared to the CNN model algorithm. Moreover, the performance of the Cascade RCNN model was similar to the gold standard developed by endoscopists. Additionally, the use of the Cascade RCNN model resulted in a reduction in detection time and a significant improvement in efficiency. The detective rates of the Cascade RCNN model, CNN model, and endoscopic detection alone in early esophageal cancer and precancerous lesions were 88.8% (71/80), 56.3% (45/80), and 44.1% (35/80), respectively [12]. Given the extensive duration required for EUS training and the intricate nature of the techniques involved, there is a growing interest among researchers in the field of EUS-AI. EUS-AI has the potential to aid novice practitioners in their training, significantly reducing the learning curve for them. Additionally, it can serve as a tool for quality control in pancreatic diseases, by providing information about the current location and suggesting the appropriate means of examination for the next step, including assisting with endoscopic ultrasound-guided fine-needle aspiration/biopsy procedures. However, the utilization of EUS in primary hospitals is relatively limited, resulting in a lack of operational experience among endoscopists. Consequently, the incorporation of EUS-AI in primary hospitals could prove highly advantageous in enhancing the overall disease detection rate. This paper aims to provide a comprehensive review of the diagnosis, prognosis, and quality control of EUS-AI in various digestive diseases such as subepithelial lesions (SELs), early esophageal cancer, early gastric cancer (EGC), and pancreatic diseases including pancreatic cystic lesions, autoimmune pancreatitis (AIP), and pancreatic cancer (PC) in the last ten years. The objective is to facilitate the advancement of this examination method in the realm of medical health. Additionally, this paper sheds light on the limitations and future prospects of EUS-AI in the field of digestive diseases (Figure 2).

## 2. Methods

### 2.1. Search Strategy

For this review we searched articles in the PubMed Database published within the past ten years (2013–2023) in order to assess the latest advancements in this area. Search terms in the title, abstract, and keywords are as follows: (“artificial intelligence” OR “AI” OR “machine learning” OR “deep learning” OR “convolutional neural network” OR “computer-assisted” OR “computer-aided” OR “neural network” OR “digital image analysis” OR “digital image processing”) AND (“endoscopic ultrasound” OR “endoscopic ultrasonography” OR “endosonography” OR “EUS”). To avoid omissions, the digestive system diseases were not included in the retrieval strategy. The data search was limited to studies written in English.

### 2.2. Inclusion and Exclusion Criteria

We carefully selected articles that accurately described the applications and prospects of EUS-AI for digestive system diseases. Studies that met all of the following inclusion criteria were selected: (1) evaluated EUS-AI for the diagnosis, prognosis, and quality control of digestive system diseases; (2) the final diagnosis was established by the histopathological diagnosis after surgical or endoscopic resection (ER) or EUS-guided fine-needle aspiration/biopsy; (3) an AI algorithm was applied to the diagnosis of patients with digestive system diseases using EUS; (4) study results demonstrated the diagnostic performance of CAD algorithms, including area under the curve (AUC), sensitivity, specificity, or accuracy, with or without EUS experts as controls.

Studies were excluded based on any of the following criteria: (1) did not evaluate digestive system diseases by EUS-AI; (2) studies were conference proceedings, abstracts, editorials, case reports, or letters; (3) duplicate articles; (4) incomplete data available.

## 3. Types of Digestive System Diseases Diagnosed and Prognosed by EUS-AI

### 3.1. Subepithelial Lesions

SELs of the gastrointestinal tract are masses or mass-like structures originating from the non-mucosal layer, including gastrointestinal stromal tumors (GISTs), leiomyoma, schwannoma, neuroendocrine tumor (NET), ectopic pancreas, lipoma, and hemangioma, of which the first two are the most common. GISTs originate from interstitial cells of Cajal or stem cells with a tendency to differentiate interstitial cells of Cajal, and they usually occur in the stomach and small intestine. GISTs have variable malignant potential, accounting for 1–3% of gastrointestinal malignancies. Surgical resection of small lesions without metastasis might be the only possible way to cure GISTs [13,14,15]. Therefore, distinguishing them from benign SELs in early stages is the key to treating GISTs.

CT and MRI are the tests more commonly used to diagnose GISTs [16]. The risk of postoperative recurrence of GISTs is related to the modified US National Institutes of Health classification. Clinically, GISTs are categorized into four grades of very low, low, intermediate, and high risk based on size, site of development, number of microscopic nuclear divisions, and whether or not it is ruptured [17]. In the very low-risk and low-risk group, GISTs are mostly rounded with well-defined borders, and the lesions on CT reveal low density or equal density; the lesions on MRI generally appear to have hypointense or isointense signals on T1-weighted imaging, slightly hyperintense signals on the T2-weighted imaging, and evenly hyperintense signals on fat suppression T2-weighted imaging. Intermediate-risk and high-risk lesions tend to be irregularly shaped with blurred borders. The lesions are shown heterogeneously in both CT and MRI. A consensus report from the German GIST Imaging Working Group suggested that MRI as an option in case of liver-specific problems or contraindications to CT [18]. CT and MRI are usually used to examine larger tumors, and EUS has become the diagnostic procedure of choice for small GISTs [19].

EUS can clearly observe the size and morphology of gastrointestinal tumors, the structure of each layer of the gastrointestinal wall, and the invasion of adjacent organs. It has become the best diagnostic modality for SELs in recent years [20]. The typical EUS imaging features of GISTs are hypoechoic solid masses originating from the fourth layer of ultrasound with well-defined borders while the structure of the fifth layer of ultrasound is clear and intact. However, it is more difficult to distinguish between GISTs and leiomyoma with EUS alone. Several studies have been conducted to input filtered high-quality EUS images as a training set into an AI model, extract meaningful image features for model construction, and then input EUS images from the validation set into the model to verify its ability to diagnose GISTs and non-GISTs. It is concluded that EUS-AI has the potential to be a good choice for diagnosing SELs (Table 1).

In 2020, Minoda et al. studied the diagnostic accuracy of EUS-AI based on gastric SELs for GISTs and non-GISTs, indicating that EUS-AI has an accurate diagnosis for GISTs ≥20 mm [21]. In 2022, Minoda et al. verified that this AI model could also be used to distinguish GISTs and non-GISTs patients from non-gastric SELs. The authors collected EUS images of 52 non-gastric SELs patients (esophagus, *n* = 15; duodenal, *n* = 26; colon, *n* = 11), and they noticed that the diagnostic accuracy of EUS-AI improved as the size of the lesion increased, independent of lesion location [22]. A recent meta-analysis (including 7 studies with 2431 patients) noted that the pooled sensitivity and specificity of EUS-AI by CNN in diagnosing GISTs were 0.92 (95%/CI, 0.89–0.95) and 0.82 (95%/CI, 0.75–0.87), respectively, which was higher than those of endoscopists. Two of the studies assessed the ability to predict the malignant potential of GISTs; the very low-risk and low-risk GISTs were classified as the low-risk group, while the intermedium-risk and high-risk GISTs were classified as high-risk group. The pooled sensitivity and specificity for diagnosing high-risk GISTs were 0.84 (95%/CI, 0.68–0.94) and 0.81 (95%/CI, 0.73–0.86), and the summary diagnostic odds ratio was 28.80 (95%/CI, 3.48–238.31), indicating that the EUS-AI model could accurately predict the malignant potential of GISTs [23].

Studies have tested the accuracy of contrast-enhanced harmonic endoscopic ultrasound (CH-EUS) using AI algorithms to diagnose SELs. Tanaka et al. retrospectively examined 53 patients with GISTs and leiomyomas to evaluate their diagnostic accuracy by using DL involving a residual neural network and leave-one-out cross-validation, combining it with the SiamMask technique to track and trim lesions in CH-EUS videos. The sensitivity, specificity, and accuracy of AI in diagnosing GISTs were 90.5%, 90.9%, and 90.6%, respectively, and those of endoscopists were 90.5%, 81.8%, and 88.7%, respectively (*p* = 0.683), indicating that the diagnosis of CH-EUS images between AI and endoscopists was comparable [24].

Notably, Hirai et al. conducted a multicenter retrospective study to develop an EUS-AI model for common SELs (GISTs, leiomyoma, schwannoma, NET, ectopic pancreas), which assessed the diagnostic accuracy of the model and endoscopists. This study was the first to combine AI and SELs EUS images for classification and recognition, and it showed that the EUS-AI had a diagnostic accuracy of 86.1% for the five SELs categories, which was significantly higher than that of endoscopists; moreover, this model had high sensitivity and accuracy in distinguishing GISTs from non-GISTs, with 98.8% and 89.3%, respectively, which was noticeably higher than that of endoscopists [25].

**Table 1:** Studies assessing the sensitivity, specificity, and diagnostic accuracy of AI models for GISTs.

Study	Study Design	AI Model	Patient Population	Research Object	Outcomes for the AI Model
Minoda et al. [21]	Retrospective (Japan)	CNN	SELs < 20 mm: Total Patients = 30 GISTs = 23 Leiomyoma = 5 Schwannoma = 1 Ectopic Pancreas = 1 SELs ≥ 20 mm: Total Patients = 30 GISTs = 24 Leiomyoma = 4 Schwannoma = 1 Ectopic Pancreas = 1	EUS Images	Recognition of GISTs in SELs < 20 mm: Sensitivity = 86.3% Specificity = 62.5% Accuracy = 86.3% AUC = 0.861 Recognition of GISTs in SELs ≥ 20 mm: Sensitivity = 83.3% Specificity = 91.7% Accuracy = 90.0% AUC = 0.965
Minoda et al. [22]	Retrospective (Japan)	CNN	Total Patients = 52 GISTs = 36 Leiomyoma = 14 Ectopic Pancreas = 1 Appendiceal Mucocele = 1	EUS Images	Recognition of GISTs: Sensitivity = 100% Specificity = 86.1% Accuracy = 94.4% AUC = 0.980
Tanaka et al. [24]	Retrospective (Japan)	DL	Total Patients = 53 GISTs = 42 Leiomyoma = 11	CH-EUS Images	Recognition of GISTs: Sensitivity = 90.5% Specificity = 90.9% Accuracy = 90.6%
Hirai et al. [25]	Retrospective (Japan)	CNN DCGAN Semi-supervised Learning	Total Patients = 631 GISTs = 435 non-GISTs = 196 (Leiomyoma = 97, Schwannoma = 33, NET = 47, Ectopic Pancreas = 19)	EUS Images	Recognition of GISTs: Sensitivity = 98.8% Specificity = 67.6% Accuracy = 89.3%

Abbreviation: AI, artificial intelligence; CNN, convolutional neural network; GISTs, gastrointestinal stromal tumors; SELs, subepithelial lesions; EUS, endoscopic ultrasound; AUC, area under the curve; DL, deep learning; CH-EUS, contrast-enhanced harmonic endoscopic ultrasound; DCGAN, deep convolutional generative adversarial network.

### 3.2. Early Esophageal Cancer

Early esophageal cancer refers to invasive carcinoma with lesions confined to the mucosal layer, regardless of whether or not regional lymph node metastasis was presented. The symptoms of early esophageal cancer are relatively insidious and usually appear in the middle to late stage of the disease. The prognosis of esophageal cancer is closely related to its staging. The five-year survival rate of patients with advanced esophageal cancer is only about 10%, while early esophageal cancer can reach 90% after surgery [26]. Therefore, the early diagnosis and early treatment of esophageal cancer are of great significance to improve the prognosis for patients. EUS is the most accurate imaging modality for T staging of esophageal cancer [27]. T stage is determined by the number of layers of the primary tumor invading the esophageal wall and the depth of adjacent tissue infiltration. Tis a high-grade dysplasia confined to the epithelium and not penetrating the lamina propria, T1a tumors invade the lamina propria or muscularis mucosae, and T1b tumors invade the submucosa [28]. The stage of the tumor determines how it will be treated, and the American College of Gastroenterology guidelines recommend endoscopic treatment for low-grade intraepithelial neoplasia, Tis, T1a, superficial low-risk T1b, and high-risk T1b esophageal adenocarcinoma with surgery, while advanced tumors required esophagectomy, with perioperative chemotherapy or chemoradiotherapy before surgical treatment [29]. This is based on the relative subjectivity of endoscopists and the variability of operation between them, which might lead to misdiagnosis of the diseases [30]. A study by Wang et al. noted that the sensitivity of EUS for T staging of esophageal cancer ranged between 0% and 70.8%, but the specificity ranged between 71.0% and 100.0%, both of which were dependent on clinical pathological stage. The overall accuracy of EUS T staging was 58.6% [31]. This has led to an exploration of whether EUS-AI could help solve the above diagnostic dilemma.

Knabe et al. developed an EUS-AI system based on a deep convolutional neural network (DCNN), which retrospectively collected 1020 EUS images from 577 patients with esophageal adenocarcinoma for training and internal validation. The results showed that AI was able to identify mucosal carcinoma (T1a) with a sensitivity of 72%, specificity of 64%, and accuracy of 68%, while the sensitivity, specificity, and accuracy of identifying submucosal carcinoma (T1b) were 31%, 78%, and 67%, respectively. The sensitivity, specificity, and accuracy of differentiating T1a and T1b were 66%, 49%, and 55%, respectively. This study has shown that AI is likely to be able to help endoscopists detect and diagnose early esophageal cancer in the future and may also provide guidance on the treatment for esophageal cancer [32].

### 3.3. Early Gastric Cancer

Gastric cancer ranks fifth in cancer incidence and fourth in mortality [33]. The invasive depth of tumor is a significant factor affecting the staging and survival of gastric cancer [34]. Accurate staging prior to treatment is crucial for the EGC. Meanwhile, the common treatment options include endoscopic submucosal dissection (ESD) or endoscopic mucosal resection (EMR) and surgery. The absolute indication for ER for EGC is a differentiated intramucosal carcinoma ≤2 cm in diameter and without ulceration; regarding the criteria for relative indication, they include (1) diameter >2 cm, intramucosal carcinoma, differentiated, without ulceration; (2) intramucosal carcinoma, differentiated, with ulceration, ≤3 cm in diameter; (3) intramucosal carcinoma, undifferentiated, without ulceration, ≤2 cm in diameter [35]. Surgical treatment is recommended when submucosal infiltration is highly suspected by preoperative evaluation [36]. So far, CT and EUS are the common imaging methods for accurate preoperative staging of gastric cancer. CT manifestations of gastric cancer present as focal or diffuse heterogeneous gastric wall thickening. EUS can visualize the entire gastric wall and has higher accuracy in distinguishing EGC from advanced gastric cancer. EUS has become the preferred tool for local staging of gastric cancer nowadays [37]. Previous studies have reported an accuracy of 67% to 72% in determining the depth of infiltration by EUS for EGC. EUS are more dependent on operators’ experience, while the prevalence of gastric cancer is closely related to the regional distribution of patients, which has led to a worldwide imbalance in the operating experience and technical expertise of endoscopists in diagnosing gastric cancer [38]; thus, EUS-AI in EGC has attracted more and more attention.

The size, ulceration, differentiation, and location of the EGC are major factors that affect the accuracy of the T staging of EUS. Kim et al. used decision tree analysis to explore factors affecting the accuracy of EUS T staging and identify factors leading to overestimation and underestimation of EGC diagnosis. The results showed that after decision tree analysis, the accuracy of EUS T staging of EGC differed greatly under different conditions, fluctuating from 34.0% to 74.6%. For lesions >3 cm, the presence of ulcers was associated with overestimation; for lesions ≤3 cm, the type of differentiation and the location of the tumor had a greater impact on EUS T staging. In well-differentiated EGC, location was the main factor affecting the accuracy of EUS T staging. EGC was easily underestimated when the diameter was less than 3 cm and the lesion was located in the upper and middle part of the stomach. The focus of this study was to predict the accuracy of EUS T staging in patients with EGC, but the accuracy of prediction by influencing factors was not particularly high, and the study did not provide answers about which patients would benefit from EUS. Considering that the AI algorithm only used EUS findings and excluded the results of gastroscopy, the researchers mentioned that it might be possible to incorporate these with the results of gastroscopy in the decision tree, which could confirm the necessity of EUS for patients in some cases, thus improving the prediction accuracy of EUS [39].

### 3.4. Pancreatic Diseases

At present, the common examinations used to diagnose pancreatic diseases in clinical practice are US, CT, MRI, positron emission tomography-computed tomography (PET/CT), and EUS. The results of them are often very similar, differing only in subtle ways, but the treatment and prognosis for different pancreatic diseases are widely divergent, which requires a high level of expertise on the part of the physician. PET/CT is a kind of test for functional activity of the lesion, which is mainly used to evaluate the efficacy of neoadjuvant chemotherapy and recurrence of tumors after surgical resection. EUS not only provides high-resolution images in real time, but also allows rapid on-site evaluation (ROSE) by endoscopic ultrasound-guided fine-needle aspiration (EUS-FNA) and endoscopic ultrasound-guided fine-needle biopsy (EUS-FNB) to predict the types of diseases and accurately characterize them when the pathology results are returned.

#### 3.4.1. Pancreatic Cystic Lesions

Pancreatic cystic lesions (PCLs) are abnormal inflammatory or proliferative lesions of the pancreas with a prevalence rate up to 42% [40,41]. Most of them are benign, but some subtypes of PCLs are highly likely to develop into malignant tumors, and their pathological features are often characterized by a mucinous phenotype. For example, intraductal papillary mucinous neoplasm (IPMN) and mucinous cystic neoplasms (MCN) and so on [42]. Early identification of PCLs has significant value for the treatment and prognosis of the diseases. However, there are limitations in identifying the types of PCLs by EUS alone, and poor interobserver agreement due to the varying levels of endoscopists for EUS. Therefore, EUS-AI is gradually coming into the limelight and has been studied by many investigators. Nguon et al. developed a CNN model that retrospectively collected EUS images from 59 MCN and 49 serous cystic neoplasms (SCN) patients, and this model allowed for the identification of two different types of isolated pancreatic cystic neoplasms. Their algorithm achieved an overall accuracy of 82.75%, which is comparable to the performance of classification by experienced endoscopists [43]. Vilas-Boas et al. focused more on AI for the classification of groups and used a high-precision algorithm of CNN for the automatic identification of mucinous pancreatic cysts. A total of 5505 EUS images were extracted, among which, 3725 depicted mucous lesions and 1780 showed non-mucous lesions. All images were divided into two data sets, namely the training data set and the validation data set. The validation data set was used to evaluate the diagnostic effectiveness of the CNN algorithm in distinguishing mucinous from non-mucinous lesions. The authors ultimately concluded that the overall accuracy, sensitivity, and specificity of the model were 98.5%, 98.3%, and 98.9%, and the AUC was 1. This study provided a timely estimate of the likelihood of lesion malignancy by distinguishing PCLs as mucinous or non-mucinous lesions, which might make the EUS-AI an important tool for risk stratification of PCLs in clinical practice, facilitating the management of patients and subsequent follow-up [44].

IPMN is the precursor to invasive PC [45]. A retrospective study using the CNN model collected static EUS images of 50 patients with IPMN to identify the diagnostic performance of malignant tumors. It showed that the accuracy of predicting IPMN as malignant tumors by AI was 94%, and its diagnostic accuracy was higher than that of conventional EUS (40–60%) and endoscopists’ diagnoses (56%) [46]. Endoscopic ultrasound-guided needle-based confocal laser endomicroscopy (EUS-nCLE), as an emerging technology, enables the confocal laser probe to enter the cystic cavity through a 19 G puncture needle to form real-time tissue microscopic imaging, which further improves the diagnostic accuracy of pancreatic cystic tumors [47,48]. Several studies have proved the feasibility of the EUS-nCLE model to differentiate PCL types [49,50,51]. Therefore, a single-center prospective study assessed the diagnostic performance of AI combined with EUS-nCLE for advanced IPMN and explored whether it could be used for risk stratification of IPMN. Machicado et al. designed two CAD algorithms based on CNN: one of the CNN-CAD systems in the overall model automatically extracted all features of nCLE and predicted high-grade dysplasia and/or adenocarcinoma (HGD-Ca); the other CNN-CAD system in the segmentation model was trained to identify segments of papillary structures, and measure the thickness and darkness of papillary epithelium to distinguish low- or intermediate-grade dysplasia (LGD) from HGD-Ca. Compared with the Fukuoka and AGA guidelines for risk stratification, this study found that the two nCLE-guided CNN-CAD algorithms had higher sensitivity and accuracy with comparable specificity in diagnosing HGD-Ca. In terms of IPMN risk stratification, the two CNN-CAD models were more accurate compared to the guidelines, although with similar specificity. This study demonstrated that the two CNN-CAD algorithms based on the n-CLE model could diagnose advanced tumors in IPMN more accurately, while being feasible and accurate in terms of risk stratification [52].

#### 3.4.2. Autoimmune Pancreatitis

AIP is an immune-mediated fibroinflammatory subtype of CP [53,54]. The typical AIP takes diffuse pancreatic enlargement as its performance, but the atypical AIP is focused on mass enlargement. Therefore, the distinction between atypical AIP (focal AIP) and pancreatic malignancies (especially PDAC) is extremely critical. Existing guidelines consider MRI and CT as important tests for the diagnosis of AIP, and EUS is mainly used to obtain cytohistological specimens despite providing a wealth of morphological features [55]. EUS-guided tissue acquisition techniques include EUS-FNA and EUS-FNB: EUS-FNA indicates that cells are aspirated from the target tissue using a conventional straight needle, and cytopathologists determine the type of lesion by observing the abnormal cells and their characteristics in the aspirated sample. However, the diagnostic accuracy of this test depends on the availability of ROSE and it is more dependent on the diagnostic experience of the cytopathologists; at the same time, EUS-FNB uses a new generation of coarse needle that allows not only cytological evaluation but also histological examination by preserving tissue structure, which makes an effective diagnosis of AIP possible. Therefore, EUS-FNB is increasingly used as an alternative to EUS-FNA [56]. Thomsen et al. examined the utility of pancreatic EUS-FNB based on a large single-center study of 852 specimens from 723 patients, which found that pancreatic EUS-FNB for AIP had an accuracy of 0.992 (95% CI 0.983–0.997). The sensitivity and specificity of EUS-FNB for AIP were 0.833 (95% CI 0.586–0.964) and 0.995 (95% CI 0.988–0.999). This suggested the promising potential advantages of EUS-FNB in diagnosing AIP [57].

The diagnostic accuracy of AIP is highly correlated with the operation of the endoscopists and the experience of the cytopathologists, while both EUS-FNA and EUS-FNB operations are invasive. Even though the incidence of adverse events is rare, there is still a risk of complications for patients. Therefore, some studies have used EUS images or EUS videos in combination with AI to investigate the diagnostic accuracy of AIP. Guo et al. conducted a retrospective study using multivariate stepwise logistic regression and receiver operating characteristics (ROC) analyses. Ninety patients with focal autoimmune pancreatitis (FAIP) and 196 patients with PC were collected and randomly divided into two groups, the derivation group and the validation group. A predictive model was constructed based on all EUS characteristics from the derivation group and its effectiveness in evaluating the two diseases was verified in the validation group. This study demonstrated that diffuse hypoechogenicity, bile duct wall thickening, and hyperechoic foci/strands were three independent predictors, with an AUC of 0.975 (95%/CI, 0.959–0.990). Considering the subjective nature of distinguishing diffuse or focal hypoechogenicity by endoscopists, the authors excluded these two characteristics and designed another prediction model by multivariate stepwise logistic regression analysis. The results showed that main pancreatic duct dilation, common bile duct dilation, bile duct wall thickening, and hyperechoic foci/strands were independent predictors, with an AUC of 0.951 (95%/CI, 0.929–0.974). According to the optimal cutoff value, the sensitivity and specificity of the model were 83.7–91.8% and 93.3–95.6% [58]. Marya et al. used static EUS images and video databases to develop the CNN model. After training, the model could analyze EUS videos in real-time and accurately distinguish AIP from PDAC and benign pancreatic diseases (CP and normal pancreas). The sensitivity and specificity of differentiating AIP and PDAC were 90% and 93%, respectively. The sensitivity and specificity to distinguish AIP from CP were 94% and 71%, respectively. The specificity between AIP and normal pancreas was 98%. The sensitivity and specificity of AIP and non-AIP were 90% and 85%, respectively [59].

#### 3.4.3. Pancreatic Cancer

PC is a highly lethal malignancy with a global five-year overall survival rate of less than 10%. It is difficult to detect while patients have mild or asymptomatic symptoms [60]. PDAC is the most common type of PC, and the majority of diseases are diagnosed at an advanced stage with poor prognosis. Early detection of small lesions and timely excision could improve the five-year survival rate to 80.4% [61]. Traditional imaging techniques such as CT and MRI may not be able to discover smaller lesions, while EUS is the most sensitive modality for the recognition of small solid pancreatic lesions, especially for lesions smaller than 20 mm [62,63]. Therefore, several studies have evaluated EUS-AI in PC settings (Table 2).

In order to distinguish PC from noncarcinomatous pancreatic lesions, Kuwahara et al. collected 22,000 EUS images of 933 patients to evaluate the diagnostic effectiveness of the AI model developed by DL. The authors found that the AUC, sensitivity, specificity, and accuracy (95%/CI) of the diagnosis of PC were 0.90 (0.84–0.97), 0.94 (0.88–0.98), 0.82 (0.88–0.92), and 0.91 (0.85–0.95), respectively. It was also indicated that the model could be utilized to distinguish PDAC, pancreatic adenosquamous carcinoma, acinar cell carcinoma, metastatic pancreatic tumor, neuroendocrine carcinoma, NET, solid pseudopapillary neoplasm, CP, and AIP [64]. As we all know, further external validation is required in the future. Tonozuka et al. developed a CAD system using EUS images and evaluated the ability of the system to discriminate PDAC from CP and normal pancreatic patients. The results presented excellent results of this model in detecting PDAC, with AUCs of 0.924 and 0.940 in the validation and test setting, respectively [11]. In addition, a systematic review of 11 studies investigating the role of EUS-AI in the diagnosis of PC found overall accuracy, sensitivity, and specificity in the range of 80–97.5%, 83–100%, and 50–99%, respectively [65].

EUS-FNA and EUS-FNB are commonly used techniques for the diagnosis of pancreatic diseases by cytohistopathology. The uneven level of practice and experience among endoscopists leads to differences in the quality of the tissue samples they obtain. To address this challenge, AI has emerged in recent years as a promising tool to improve the accuracy and efficiency of EUS-guided tissue sampling. AI potentially assists EUS-FNA/FNB through real-time feedback obtained by the endoscopists during the procedure, helping to select the appropriate type and size of the puncture needle, guiding the optimal location and depth of the puncture, and providing feedback on the quality of the sample obtained. Thus, AI can reduce the number of punctures required to obtain an adequate sample, improve puncture accuracy, and minimize the risk of complications [66]. In addition, with the innovation of image recognition by AI and the development of cytopathology, some researchers have applied AI to analyze pathological specimens by EUS-FNA/FNB. Zhang et al. employed EUS-FNA to perform biopsies in a PC group and a non-PC (mild atypical lesions, other tumors, or no tumor) group, and applied ROSE on detected specimens after staining. Internal testing and external validation were conducted on pathological stained sections by the deep convolutional neural network (DCNN) system. Both the AUCs of internal testing and external validation were >0.9, which was comparable to the diagnostic ability of cytopathologists. Considering that some hospitals might be short of cytopathologists, the diagnostic accuracy of endoscopists who have received standardized training in pathology was compared with the DCNN model, and the results showed that the sensitivity of the DCNN model was higher than that of endoscopists [67]. Ishikawa et al. established that the DL model’s diagnostic accuracy of unstained biopsy specimens of pancreatic diseases was lower than that of macroscopic on-site evaluation (MOSE). Nevertheless, the diagnostic accuracy of the AI model after specimen staining was comparable to that of MOSE. The sensitivity, specificity, and accuracy of MOSE after staining were 88.97%, 53.5%, and 83.24%, respectively. Comparatively, the sensitivity, specificity, and accuracy of AI-assisted EUS-FNB were 90.34%, 53.5%, and 84.39%, respectively [68].

EUS has numerous modes, including B-mode, CH-EUS, endoscopic ultrasound-elastography (EUS-EG), etc. CH-EUS and EUS-EG could be used as complementary tools to characterize focal pancreatic lesions. Even though CH-EUS could not improve the detection rate of lesions, it could assist in the antidiastole of the diseases [69,70,71,72,73]. CH-EUS, which uses contrast agents combined with tissue harmonic imaging to depict the microvascular system in real time, has shown promise in differentiating benign from malignant pancreatic masses [74]. The steep learning curve of EUS requires the operators to be skilled in human anatomy and manipulation [75], thus creating an urgent need for new techniques that can emerge to objectively identify and classify CH-EUS images to assist in diagnosis. In addition, based on the temporal change in echo enhancement intensity, a time-intensity curve (TIC) can be generated. There is evidence that CH-EUS using TIC analysis is very effective in differentiating various pancreatic lesions [76]. Tang et al. constructed a novel AI-assisted diagnostic system (CH-EUS MASTER) based on DCNN and RF algorithms and applied it to two models by retrospectively collecting images or videos of CH-EUS to achieve: (1) identification and tracking of pancreatic masses dynamically in real time; (2) differentiation between PC and CP by TIC analysis. The results showed that the average overlap rate of model 1 was 0.708 with an accuracy of 87.8% at the image overlap threshold of 0.50, compared to manual annotation by endoscopists. Model 2 identified PC with an accuracy of 88.9%. This system is a promising AI system for diagnosing malignant and benign pancreatic masses [77]. EUS-EG is a diagnostic approach based on tissue stiffness measurement. Săftoiu et al. conducted a prospective, blinded, multicentric study using AI-assisted EUS-EG to discriminate between CP and PC, and the authors observed that the model had a training accuracy of 0.9114 (95% CI 0.8987–0.9242) and a test accuracy of 0.8427 (95% CI 0.8309–0.8544). The sensitivity, specificity, PPV, and NPV of AI-assisted EUS-EG were 0.88, 0.83, 0.96, and 0.57, respectively. This study suggests that ANN can provide a rapid and accurate diagnosis of pancreatic malignancies [78].

**Table 2:** Studies assessing the sensitivity, specificity, and diagnostic accuracy of AI models for PC.

Study	Study Design	AI Model	Patient Population	Research Object	Outcomes for the AI Model
Kuwahara et al. [64]	Retrospective (Japan)	DL	Total Patients = 694 PC = 524 Non-Cancer Patients = 170 (PDAC = 518, PASC = 5, ACC = 1, MPT = 8, NEC = 6, NET = 57, SPN = 6, CP = 58, AIP = 35)	EUS Images	Recognition of PC: Sensitivity = 94% Specificity = 82% Accuracy = 91% AUC = 0.90
Tonozuka et al. [11]	Retrospective (Japan)	CNN	Total Patients = 139 PDAC = 76 CP = 34 NP = 29	EUS Images	Recognition of PC: Sensitivity = 92.4% Specificity = 84.1% AUC = 0.940
Goyal et al. [65]	Systematic Review (United States)	ANN CNN SVM	Total Patients = 2292 PC = 1409 Non-Cancer Patients = 883	EUS Images EUS Videos EUS-EG	Recognition of PC: Sensitivity = 83–100% Specificity = 50–99% Accuracy = 80–97.5%
Zhang et al. [67]	Retrospective (China)	DCNN	Total Patients = 194 PC = 110 Non-Cancer Patients = 84	Staining EUS-FNA Specimens	Recognition of PC: Sensitivity = 92.8–94.4% Specificity = 87.5–97.1% Accuracy = 91.2–95.8% AUC = 0.948–0.976
Ishikawa et al. [68]	Retrospective (Japan)	Contrastive Learning (Unsupervised Learning)	Total Patients = 97 PDAC = 66 MFP = 13 AIP = 11 Pancreatic Neuroendocrine Tumor = 3 MPT = 3 IPMC = 1	Staining EUS-FNB Specimens	Recognition of Pancreatic Diseases: Sensitivity = 90.34% Specificity = 53.5% Accuracy = 84.39%
Tang et al. [77]	Prospective (China)	Model 1: DCNN Model 2: RF Algorithm	Total Patients in Model 1 = 950 PC = 760 Benign Pancreatic Masses = 190 Total Patients in Model 2 = 295 PC = 167 Pancreatitis = 128	Model 1: CH-EUS Images Model 2: CH-EUS Videos	Recognition of Pancreatic Diseases in Model 1: the Average Overlap Rate = 0.708; Accuracy = 87.8% Recognition of Pancreatic Diseases in Model 2: Sensitivity = 100% Specificity = 75% Accuracy = 88.9%
Săftoiu et al. [78]	Prospective (Europe)	ANN	Total Patients = 258 PC = 211 CP = 47	Hue Histogram Data Extracted from Dynamic Sequences of EUS-EG	Recognition of Pancreatic Diseases: Sensitivity = 87.59% Specificity = 82.94% Accuracy = 84.27%

Abbreviation: AI, artificial intelligence; DL, deep learning; PC, pancreatic cancer; PDAC, pancreatic ductal adenocarcinoma; PASC, pancreatic adeno-squamous carcinoma; ACC, acinar cell carcinoma; MPT, metastatic pancreatic tumors; NEC, neuroendocrine carcinoma; NET, neuroendocrine tumors; SPN, solid pseudo papillary neoplasms; CP, chronic pancreatitis; AIP, autoimmune pancreatitis; EUS, endoscopic ultrasound; AUC, area under the curve; CNN, convolutional neural network; NP, normal pancreas; ANN, artificial neural network; SVM, support vector machine; EUS-EG, endoscopic ultrasound elastography; DCNN, deep convolutional neural network; EUS-FNA, endoscopic ultrasound-guided fine-needle aspiration biopsy; MFP, mass-forming pancreatitis; IPMC, intraductal papillary mucinous carcinoma; EUS-FNB, endoscopic ultrasound-guided fine-needle biopsy; RF, random forest; CH-EUS, contrast-enhanced harmonic endoscopic ultrasound.

## 4. EUS-AI in Quality Control

In addition to building CAD models from EUS images, EUS videos, and cytohistological smears after training to improve the diagnostic accuracy of benign and malignant diseases of the pancreas, EUS-AI can also carry out quality control of the pancreatic scan process to solve the problem of missed diagnosis of diseases due to the blind area of the field of view. Existing studies have established a pancreatic scanning system with EUS as the standard procedure guided by systematic scanning in separate stations [79,80]. However, the complex anatomical structure of EUS for diseases increases the hardship of the interpretation of images. For this reason, Zhang et al. developed a system called BP MASTER to create a station classification model and a segmentation model, which were then subjected to internal and external verification. The classification model was utilized to determine the current scan site and guide the operation of the next site, while the segmentation model focused on monitoring the pancreas/abdominal aorta/portal confluence in real-time. If the pancreas and important blood vessels continued to disappear, it was recommended to return to the previous station for rescanning. The researchers found that the accuracy of the classification model for site identification during internal and external verification was 94.2% and 82.4%, respectively. The mean F1 index (Dice) for the segmentation model was 0.836 and 0.715. Additionally, the researchers extracted 396 video clips and applied them to the station classification model, achieving a per-frame accuracy of 86.2%. Moreover, the BP MASTER system was shown to reduce the learning curve for inexperienced students using EUS-AI to identify PC. On the basis of a previous study, a prospective study was conducted involving eight students with one year of experience in gastroenterology. The students had not participated in operation training for EUS. The trainees’ recognition accuracy of processed EUS videos significantly increased from 67.2% to 78.4% (95%/CI, 0.058–1.663; *p* < 0.01) [75].

## 5. Discussion and Prospects

In summary, the diagnostic accuracy of EUS-AI for digestive system diseases is comparable to or even better than that of endoscopists. EUS, with its high-resolution imaging, can effectively observe the lesion site and provide a diagnosis advantage over other imaging tests such as US, CT, MRI, and PET/CT. Nevertheless, the diagnostic ability of endoscopists is highly correlated with their knowledge reserve, clinical experience, and operation proficiency. With the increase in operation times, endoscopists may miss the diagnosis of diseases due to fatigue or inattention. Manual diagnosis by endoscopists is also subjective. EUS-AI, which combines AI with EUS examinations, enables early diagnosis of disease while accelerating the treatment process and improving patient prognosis. By implementing quality control during the inspection process, EUS-AI could alleviate the overall medical burden on individuals and healthcare systems worldwide. Moreover, the addition of AI can shorten the learning curve for novice doctors, who typically require extensive training by experienced endoscopists to standardize their EUS skills. Consequently, these discoveries may eliminate the “automation bias” held by some individuals towards EUS-AI, representing a significant advancement for CAD in clinical diagnosis.

In recent years, there has been a growing interest among researchers in the use of EUS-FNA/FNB instead of postoperative cytopathology and histopathology. AI-assisted EUS-FNA/FNB has shown promising results, as it allows for the precise localization of lesions and avoids important vessels during puncture. By optimizing the sampling site, angle, and number of times, the prognostic risk for patients can be significantly reduced. Additionally, to address the issue of missed diagnosis resulting from the blind field of view during operations, some studies have utilized EUS-AI to create pancreatic segmentation and classification models. These models provide real-time information about the current location and guide subsequent examination methods, representing a relatively novel and valuable tool. If this technique is applied to examine other digestive diseases, this could be a major step forward in the field of disease screening.

The applications of AI in EUS come with certain limitations. Firstly, most of the current studies are retrospective single-center studies, which means that the data sources in the obtained datasets lack universal representation. As a result, models built on such data might be prone to information bias. Secondly, anonymized or de-identified data often need to be traced back to the patient for diagnosis and treatment. This introduces a potential risk of data breaches and unauthorized access to patient information. Thirdly, the standardization of data collection and data analysis is insufficient. In order to ensure accurate diagnosis and broad applicability, standardized processing of data acquisition and analysis should be established. Fourthly, the advent of EUS-AI, a machine–human collaborative examination, has brought about a significant transformation in the conventional doctor–patient relationship. The responsibility for any misdiagnosis resulting from the use of AI falls not only on the doctors but also on the model developers and software platform vendors involved. Active and flexible laws and regulations are still relatively lacking. Fifthly, the particularly specifical limitation of AI is the “black box problem” [81], that is, only the input layers and output layers are visible, and the operation and recognition in the hidden layers are opaque. This makes it difficult for doctors and model developers to explain the reasons for potential biases, errors, and unintended consequences, posing a great challenge in the context of evidence-based medicine.

In the information age, emerging technologies such as AI are still at an early stage. While there are limitations in the diagnosis of diseases through EUS-AI, researchers can take certain steps to enhance its application in clinical settings. In the future, AI algorithms should be refined to create visualized AI decision-making processes. Additionally, large-sample, multi-center prospective studies should be conducted to cover a range of diseases. The incorporation of high-quality images or videos should be maximized, and integrated models should be flexibly applied. Furthermore, an open, quality-monitored data collection server should be established to enable global sharing, while ensuring data confidentiality to protect patient privacy. Clear accountability policies need to be developed to regulate AI technology effectively, ensuring its reasonable and legal application, and minimizing or avoiding harm caused by AI errors. It is important to note that EUS-AI exhibits great potential in healthcare, but it does not imply that endoscopists will be replaced by AI. Instead, the collaboration between the two can lead to more accurate decision-making in the diagnosis and treatment process, thus improving the efficacy of disease diagnosis and facilitating the further development of individualized precision medicine.

## Data Availability

Not applicable.
